# Does individualized guided selection of antiplatelet therapy improve outcomes after percutaneous coronary intervention? A systematic review and meta-analysis

**DOI:** 10.1016/j.amsu.2022.103964

**Published:** 2022-06-18

**Authors:** Naser Yamani, Samuel Unzek, Muhammad Hasnain Mankani, Talal Almas, Adeena Musheer, Humera Qamar, Shausha Farooq, Waqas Shahnawaz, Kaneez Fatima, Vincent Figueredo, Farouk Mookadam

**Affiliations:** a Department of Medicine, John H Stroger Jr. Hospital of Cook County, Chicago, IL, USA; b Department of Cardiac Imaging, Banner University Medical Centre, Phoenix, AZ, USA; c Department of Medicine, Aga Khan University Hospital, Karachi, Pakistan; d Department of Medicine, RCSI University of Medicine and Health Sciences, Dublin, Ireland; e Department of Medicine, Khaja Bandanawaz Institute of Medical Sciences Gulbarga, India; f Department of Medicine, Ziauddin University Hospital, Karachi, Pakistan; g Department of Medicine, Dow University of Health Sciences, Karachi, Pakistan; h Department of Cardiology, St.Mary Medical Center, Langhorne, PA, USA; i Department of Cardio Oncology, Banner University Medical Centre, Phoenix, AZ, USA

**Keywords:** Antiplatelet, Percutaneous, Mortality, Cardiovascular

## Abstract

**Background:**

The potential benefits of individualized guided selection of antiplatelet therapy over standard antiplatelet therapy in improving outcomes in patients undergoing percutaneous coronary intervention (PCI) have not been established. Therefore, we pooled evidence from available clinical trials to assess the effectiveness by comparing the two regimens in patients undergoing PCI.

**Methods:**

We queried two electronic databases, MEDLINE and Cochrane CENTRAL, from their inception to April 20, 2021 for published randomized controlled trials in any language that compared guided antiplatelet therapy, using either genetic testing or platelet function testing, versus standard antiplatelet therapy in patients undergoing PCI. The results from trials were presented as risk ratios (RRs) with 95% confidence intervals (CIs) and were pooled using a random-effects model.

**Results:**

Eleven eligible studies consisting of 18,465 patients undergoing PCI were included. Pooled results indicated that guided antiplatelet therapy, compared to standard therapy, was associated with a significant reduction in the incidence of MACE [RR 0·78, 95% CI (0·62–0·99), P = 0·04], MI [RR 0·73, 95% CI (0·56–0.96), P = 0·03], ST [RR 0·66, 95% CI (0·47–0.94), P = 0·02], stroke [RR 0·71, 95% CI (0·50–1.00), P = 0·05], and minor bleeding [RR 0·78, 95% CI (0·66–0.91), P = 0·003].

**Conclusions:**

Individualized guided selection of antiplatelet therapy significantly reduced the incidence of MACE, MI, ST, stroke, and minor bleeding in adult patients when compared with standard antiplatelet therapy. Our findings support the implementation of genetic and platelet function testing to select the most beneficial antiplatelet agent.

## Highlights

• Benefits of individualized guided selection of antiplatelet therapy over standard antiplatelet therapy in improving outcomes in patients undergoing percutaneous coronary intervention (PCI) have not been established.
• Guided therapy, consisting of either genetic testing or platelet function testing, can identify patients with this genetic variation allowing clinicians to provide modified and alternative treatment strategies and prescribe optimal antiplatelet agents.
• Individualized guided selection of antiplatelet therapy reduced the incidence of MACE, MI, ST, stroke, and minor bleeding in adult patients when compared with standard antiplatelet therapy.
• Can be more cost-effective than medication cost for every stroke, in-stent thrombosis, or MI case that needs hospitalization requiring more imaging and tests which can increase the total cost of treatment.

## 1 Introduction

The protocol followed for the prevention of ischemic events in patients undergoing percutaneous coronary intervention (PCI) with drug-eluting stent (DES) implantation includes dual antiplatelet therapy, consisting of aspirin and a P2Y_12_ inhibitor, as recommended by the current European Society of Cardiology (ESC) guidelines to be prescribed for a minimum of 12 months following PCI [1,2]. Out of the three major P2Y_12_ inhibitors currently available, clopidogrel is the most widely used agent because of its low cost, easy availability, and robust history of utility [[3], [4], [5]]. However, recent evidence suggests that a category of patients do not respond adequately to this medication, resulting in increased on-treatment platelet reactivity (HPR) and rates of atherothrombotic events [[4], [5], [6]]. A major factor of variable clopidogrel efficacy includes a loss-of-function genetic variation in the cytochrome P450 2C19 (*CYP2C19)* gene, which plays a role in altering clopidogrel into its biologically active metabolite, which then irreversibly binds to the P2Y_12_ receptor thus inhibiting ADP-induced platelet aggregation [7,8]. In recent years, alternative P2Y_12_ receptor inhibitors, prasugrel and ticagrelor, have been developed and have been investigated to be superior to clopidogrel in terms of platelet inhibition and reduction of major adverse cardiovascular events, but are associated with a higher risk of bleeding complications [5,[9], [10], [11]].

An individualized approach to treat patients who do not respond to clopidogrel with more potent drugs can prove to be a valid strategy to increase patient safety and reduce healthcare costs. Therefore, it is of high interest to identify patients who are non-responders to antiplatelet drugs and then to tailor their therapy to the most effective individual option [5,12]. Guided therapy, consisting of either genetic testing or platelet function testing, can identify patients with this genetic variation allowing clinicians to provide modified and alternative treatment strategies and prescribe optimal antiplatelet agents.

The current American College of Cardiology Foundation PCI guidelines state that genetic testing may be considered in high-risk patients, but current evidence is insufficient to recommend routine genetic testing in patients undergoing PCI [13]. This testing hesitancy may be due to insignificant results from previous studies comparing guided versus standard antiplatelet therapy in patients specifically with HPR or carriers of *CYP2C19* loss-of-function alleles [14,15]. However, more recent studies have investigated the implementation of genetic testing or platelet function testing as a strategy to help guide antiplatelet therapy, in patients undergoing PCI. Nonetheless, these studies lack statistical power for efficacy outcomes and report unclear results. Therefore, we performed a quantitative systematic review and meta-analysis to assess the safety and efficacy of guided versus standard selection of antiplatelet therapy in patients undergoing PCI. Through subgroup analyses, we noted if results were affected based on the type of test used (genetic *vs* platelet function testing) and the strategy employed (escalation *vs* de-escalation).

## 2 Methods

### 2.1 Data sources and search strategy

This meta-analysis was performed according to the preferred reporting items for systematic reviews and meta-analyses (PRISMA) and the Cochrane Collaboration guidelines [16,17] An electronic search of MEDLINE and Cochrane CENTRAL was conducted from their inception to April 20, 2021 without any language restrictions for articles that sought to compare clinical outcomes between guided versus standard selection of antiplatelet therapy in patients undergoing PCI. We also manually screened the reference lists of the retrieved trials, previous meta-analyses, and review articles to identify any relevant studies. Mesh terms along with Boolean operators were used to produce a search strategy for each database (Supplementary Table 1).

### 2.2 Study selection

The following eligibility criteria were used to select studies: (a) published and unpublished randomized controlled trials with a follow-up duration of at least 6 months; (b) adults (≥18 years of age) undergoing PCI; (c) guided antiplatelet therapy (consisting of genetic testing or platelet function testing), that were compared with standard antiplatelet therapy; and (d) at least one major adverse cardiovascular event or bleeding being reported. Both strategies of either escalation, which refers to switching from clopidogrel to ticagrelor, prasugrel, or double dose clopidogrel, or adding cilostazol, or de-escalation, which refers to switching from ticagrelor or prasugrel to clopidogrel, could be characterized as guided therapy.

### 2.3 Data extraction and assessment of study quality

All articles retrieved from the systematic search were exported to Endnote Reference Manager (Version X4; Clarivate Analytics, Philadelphia, PA, USA), where duplicates were identified and removed. The remaining articles were carefully evaluated by two independent reviewers (NY and MHM), and only those trials that met the previously defined criteria were selected. All trials were initially short-listed based on title and abstract, after which the full article was reviewed to affirm relevance. A third investigator (FM) was consulted to resolve any discrepancies. From the finalized trials, the following outcomes were extracted: major adverse cardiovascular events (MACEs); cardiovascular mortality; all-cause mortality; myocardial infarction (MI); stent thrombosis (ST); stroke; major bleeding, and minor bleeding. The modified Cochrane Collaboration's risk of bias tool for randomized controlled trials was used to assess the quality of published trials [18]. Upon assessment using the AMSTAR 2 appraisal tool, this systematic review was noted to have a partial level of compliance [19].

### 2.4 Statistical analysis

Review Manager (Version 5.3. Copenhagen: The Nordic Cochrane Centre, The Cochrane Collaboration, 2014) was utilized for all statistical analyses. The results from trials were presented as risk ratios (RRs) with 95% confidence intervals (CIs) and were pooled using a random-effects model. Forest plots were created to visually assess the results of pooling the results. Two subgroup analyses were carried out, according to the type of test used to guide the selection of therapy *(*genetic testing *vs* platelet function testing), and the type of strategy used for guided therapy (escalation *vs* de-escalation). The chi-square test was performed to assess for differences between the subgroups. Heterogeneity across studies was evaluated using Higgins I^2^ statistics (I^2^ = 25%–50% was considered mild, 50%–75% moderate, and >75% severe heterogeneity). Begg's test and a visual inspection of the funnel plot were conducted to evaluate the publication bias. A P value of less than 0.05 was considered significant in all cases.

## 3 Results

### 3.1 Literature search results

**Fig. 1:**
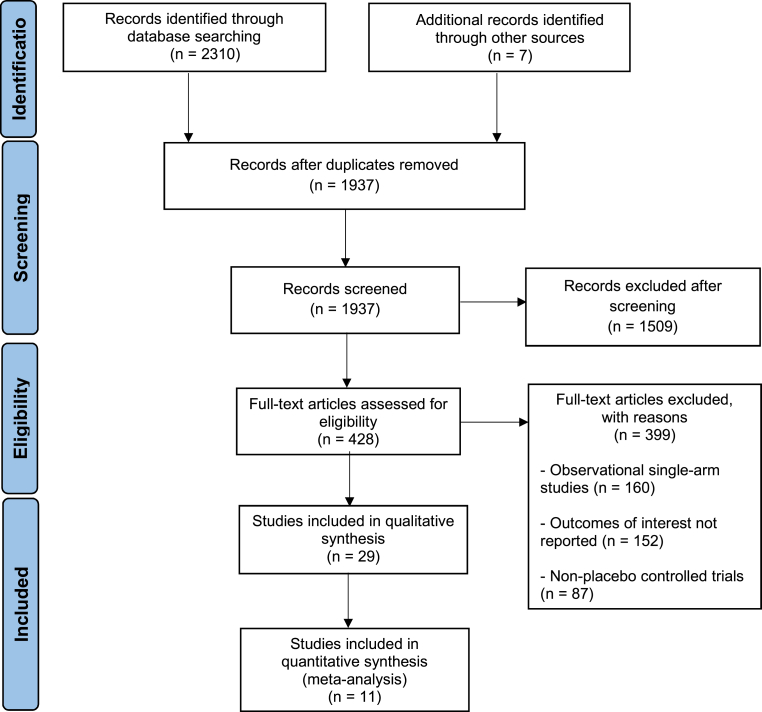
PRISMA flow diagram.

An initial search of the two electronic databases yielded 2317 potentially relevant articles. After the removal of duplicates and studies with irrelevant data, the remaining 1937 articles were screened. After exclusions, eleven trials remained for analysis [[20], [21], [22], [23], [24], [25], [26], [27], [28], [29], [30]]. The escalation strategy was employed in seven studies while de-escalation was used in four studies. Treatments undertaken in patients undergoing guided therapy included cilostazol alongside dual antiplatelet therapy in two studies, double dose clopidogrel in three studies, and ticagrelor or prasugrel in the remaining six studies. The PRISMA flow chart (Fig. 1) summarizes the results of our literature search.

### 3.2 Study characteristics and quality assessment

Eleven randomized controlled trials with a total of 18,465 PCI patients were included in the analysis. The follow-up duration ranged from 24 weeks to 64 weeks. Genetic testing was used in five studies and platelet function testing was used in six studies. Five of the eleven included trials achieved a “high” quality rating (Supplementary Table 2). We observed a symmetrical funnel plot (Supplementary Fig. 1) demonstrating minimal publication bias. Baseline characteristics of the included trials are given in Table 1.

**Table 1:** Baseline clinical features and clinical presentation of patients included in the meta-analysis.

Study name	Group	Age (mean, SD or median (Q1- Q3)	Women, n (%)			Diabetes, n (%)	Hypertension, n (%)	Active smoking, n (%)	Dyslipidemia, n (%)		Family history of CAD, n (%)		Previous ACS, n (%)	Previous PCI, n (%)		Previous CABG, n (%)	Previous stroke or TIA, n (%)
POPular Genetic [20]	Standard	61.4(11.5)	309(24)			138(11.1)	511(41)	565(45.8)	255(20.5)		467(39.5)		87(7)	91(7.3)		22(1.8)	NA
	Guided	61.9(11.1)	317(25.5)			138(11.1)	511(41)	565(45.8)	255(20.5)		467(39.5)		97(7.8)	99 (8)		47(7)	NA
TAILOR PCI [21]	Standard	62 (21–93)	645(24)			695(26)	1667(63)	637(24)	1384(53)		NA		371(14)	612(23)		188(7)	76(3)
	Guided	62 (26–95)	648(25)			733(28)	1636(62)	648(25)	1363(52)		NA		387(15)	612(23)		196(7)	72(3)
ARCTIC [22]	Standard	63	247(20.1)			449(36.6)	745(60.7)	292(23.8)	835(68.1)		NA		384(31.3)	545(44.4)		86(7)	NA
	Guided	63	223 (18.4)			440 (36.3)	776 (64)	311 (25.6)	817 (67.4)		NA		351 (28.9)	505 (41.6)		75 (6.2)	NA
ANTARCTIC [23]	Standard	81 (78–84)	180(41)			124(28)	318(72)	38 (9)	242(55)		NA		66(15)	113(26)		23(5)	NA
	Guided	80 (77–84)	164(38)			123(28)	313(72)	37 (9)	230(53)		NA		84(19)	106(24)		29(7)	NA
TROPICAL-ACS [24]	Standard	58.5(10.2)	1023(22)			287(22)	806(62)	591(45)	529(41)		466(36)		153(12)	186(14)		46(4)	NA
	Guided	59(10.1)	1029(21)			240(18)	793(61)	591(45)	546(42)		419(32)		140(11)	173(13)		39(3)	NA
PHARMCLO [25]	Standard	70.7(12.1)		130(440)	122(27.7)		329(74.8)	108(24.6)		232(52.7)	102(23.2)	95(21.6)		88(20)	37(8.4)		28(6.4)
	Guided	71.1(12.3)		153(34.2)	113(25.2)		331(73.9)	92(20.5)		251(56)	96(21.4)	96(21.4)		81(18.1)	43(9.6)		35 (7.8)
IAC-PCI [26]	Standard	57.8(10.3)		72(24.1)	97(32.4)		171(57.2)	NA		NA	NA	NA		NA	NA		NA
	Guided	57.9(10.7)		60(19.9)	92(30.6)		161(53.5)	NA		NA	NA	NA		NA	NA		NA
Zhu et al. [27]	Standard	60.1(10.9)		53(35)	33(21.9)		69(45.7)	NA		86(57)	NA	13(8.6)		NA	NA		NA
	Guided	60.2(10.9)		51(33)	27(17.5)		64(41.6)	NA		79(51.3)	NA	15(9.7)		NA	NA		NA
PATH-PCI [28]	Standard	58.34(10.2)		205(18)	330(29)		657(57.7)	573(50.3)		NA	159(14)	NA		NA	NA		NA
	Guided	58.04(10.7)		176(15)	316(27.6)		642(56)	597(52.1)		NA	134 (11.7)	NA		NA	NA		NA
Tuteja et al. [29]	Standard	62.9(10.2)		66(26)	89(35)		199(78)	37(15)		113(44)	NA	67(26)		83(58)	36(14)		15(6)
	Guided	63(9.7)		68(27)	79(31)		190(76)	28(11)		112(45)	NA	63(25)		83(61)	32(13)		7 (3)
Hazarbasanov et al. [30]	Standard	64(9.8)		30(32)	24(25.3)		87(91.6)	43(45.3)		68(71.6)	NA	28(29.5)		28(29.5)	NA		NA
	Guided	65(8.7)		36(37)	24(24.7)		83(85.6)	46(47.4)		61(62.9)	NA	26(26.8)		25(25.8)	NA		NA

ACS, acute coronary syndromes; CABG, coronary artery bypass graft; CAD, coronary artery disease; NA, not available; PCI, percutaneous coronary intervention; TIA, transient ischemic attack.

### 3.3 Results of meta-analysis

The summarized results of our meta-analysis are presented in Fig. 2. Detailed forest plots, outlining the effect size of each study, are provided in the supplementary file (Supplementary Figs. 2–9).

**Fig. 2:**
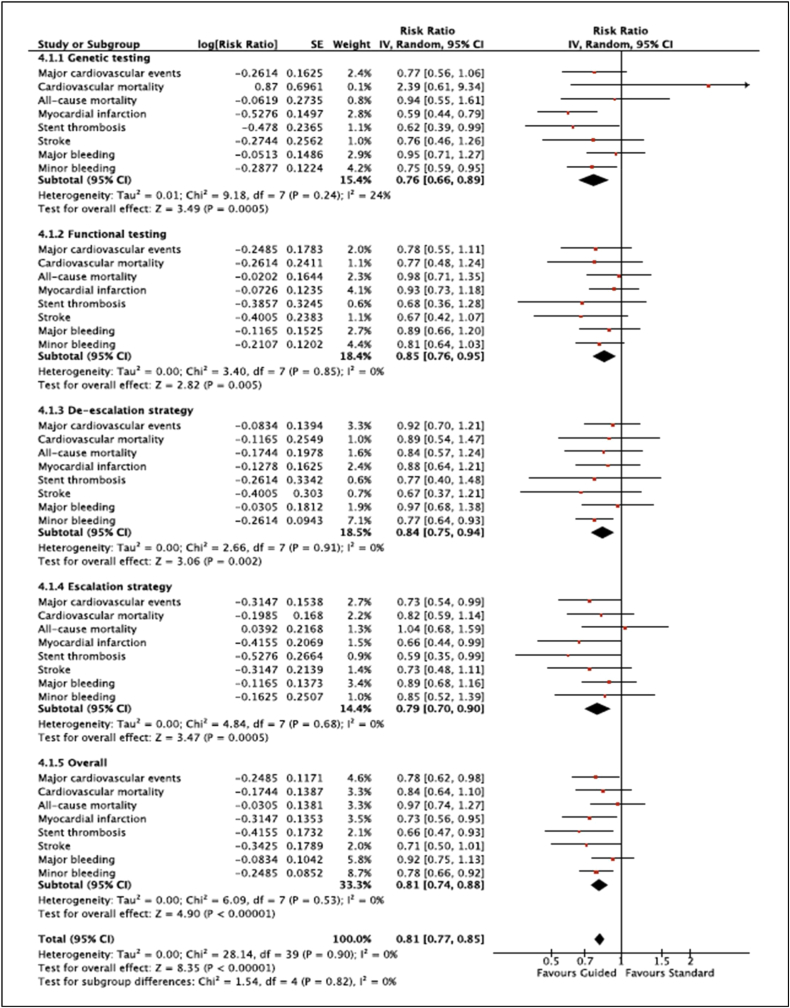
Forest plot displaying summarized results of the effect of guided vs standard antiplatelet therapy on all the outcomes assessed in this meta-analysis.

### 3.4 Major adverse cardiovascular events

All eleven included trials reported MACE. Our meta-analysis indicated that guided selection of antiplatelet therapy was associated with a significant reduction in the incidence of MACE [RR 0·78, 95% CI (0·62–0·99), P = 0·04, *I*^2^ = 77%] compared with standard antiplatelet therapy (Supplementary Fig. 2).

### 3.5 Cardiovascular *mortality*

Ten of the eleven included trials reported cardiovascular mortality. No significant difference was noted in the occurrence of cardiovascular mortality [RR 0·84, 95% CI (0·64–1.10), P = 0·21, *I*^2^ = 0%] when guided therapy was compared with standard antiplatelet therapy (Supplementary Fig. 3).

### 3.6 All-cause mortality

Eight of the eleven included trials reported all-cause mortality. No significant difference was noted in the occurrence of all-cause mortality [RR 0·97, 95% CI (0·74–1.27), P = 0·82, *I*^2^ = 0%] when guided therapy was compared with standard antiplatelet therapy (Supplementary Fig. 4).

### 3.7 Myocardial *infarction*

All eleven included trials reported MI. Our meta-analysis indicated that guided selection of antiplatelet therapy was associated with a significant reduction in the incidence of MI [RR 0·73, 95% CI (0·56–0.96), P = 0·03, *I*^2^ = 60%] compared with standard antiplatelet therapy (Supplementary Fig. 5).

### 3.8 *Stent* thrombosis

All eleven included trials reported ST. Our meta-analysis indicated that guided selection of antiplatelet therapy was associated with a significant reduction in the incidence of ST [RR 0·66, 95% CI (0·47–0.94), P = 0·02, *I*^2^ = 1%] compared with standard antiplatelet therapy (Supplementary Fig. 6).

### 3.9 Stroke

All eleven included trials reported stroke. Our meta-analysis indicated that guided selection of antiplatelet therapy was associated with a significant reduction in the incidence of stroke [RR 0·71, 95% CI (0·50–1.00), P = 0·05, *I*^2^ = 0%] compared with standard antiplatelet therapy (Supplementary Fig. 7).

### 3.10 Major *bleeding*

Nine of the eleven included trials reported major bleeding. No significant difference was noted in the occurrence of major bleeding [RR 0·92, 95% CI (0·75–1.13), P = 0·43, *I*^2^ = 0%] when guided therapy was compared with standard antiplatelet therapy (Supplementary Fig. 8).

### 3.11 Minor *bleeding*

Seven of the eleven included trials reported minor bleeding. Our meta-analysis indicated that guided selection of antiplatelet therapy was associated with a significant reduction in the incidence of minor bleeding [RR 0·78, 95% CI (0·66–0.91), P = 0·003, *I*^2^ = 0%] compared with standard antiplatelet therapy (Supplementary Fig. 9).

There was no significant difference in results between the group of studies using genetic testing for guided therapy relative to those using platelet function testing (P interaction = 0.28, *I*^2^ = 13.4%). Moreover, there was no significant difference in results between the group of studies using the escalation strategy for guided therapy relative to those using the de-escalation approach (P interaction = 0.56, *I*^2^ = 0%).

## 4 Discussion

Our meta-analysis of 18,465 patients undergoing PCI demonstrated that relative to standard antiplatelet therapy, guided selection of antiplatelet therapy was associated with a significant reduction in the incidence of MACE, MI, ST, stroke, and minor bleeding. Our findings support the implementation of genetic testing and platelet function testing in patients undergoing PCI to modify the selection of antiplatelet agents, maximizing patient safety and reducing costs.

Observational studies were excluded from our analysis due to the increased risk of confounding and selection bias that they entail; this is prevented in RCTs through randomization and blinding. Moreover, a substantial number of accurate clinical trials were available proving causality. Previous individual trials, due to their limitations and small sample sizes, had not been successful in indicating a significant benefit in the implementation of guided selection of antiplatelet therapy; a large, randomized trial examining a genotype-guided selection of antiplatelet therapy, the TAILOR-PCI (Tailored Antiplatelet Therapy Following PCI) trial, did observe a 34% relative reduction in ischemic events when carriers of *CYP2C19* loss-of-function alleles were treated with ticagrelor relative to clopidogrel, but it failed to reach statistical significance [21]. Through the pooling of results and a large sample size, this meta-analysis was successful in demonstrating significant reductions in severe, adverse outcomes by the implementation of guided antiplatelet therapy. More recently, the pooling of multiple well-controlled and blinded randomized trials show greater predictive power and efficacy than a single trial, and our study indicates similar findings.

In recent years, several studies have focused on employing guided therapy in patients undergoing PCI to help prescribe optimal antiplatelet agents with the intent of maximizing patient safety and efficacy whilst reducing costs. Compared to genotype testing, platelet function testing is more manageable and cost-effective, providing quick results determining responses to antiplatelet therapy in real-time [31]. It was observed in the PATROL study that for patients with HPR, identified by platelet function testing, switching clopidogrel to ticagrelor could significantly improve 1-year clinical outcomes without increasing the risk of bleeding [32]. However, platelet function testing has certain limitations given that it requires patients to be on treatment to define responsiveness [31]. On the other hand, genetic variations alone might be of restricted precision to identify patients with HPR status. Hence, routine implementation of these two methods is not recommended by current guidelines due to the uncertainty of these medical approaches in improving clinical outcomes and the lack of statistical power of available studies due to their limitations, such as the difficulty in determining whether the prescription of clopidogrel in the treatment plan of *CYP2C19* loss-of-function allele carriers was due to the physician failing to consider genetic test results or because of other clinical factors such as the increased risk of bleeding. We believe through our meta-analysis, the effects of such limitations are reduced and the advantages of guided selection of antiplatelet therapy are evident.

We must note that multiple factors, including electronic health record support, genotyping services available within the clinical facility, and reduced logistic encumbrances, allowed for successful implementation of genetic testing in these included studies. However, it is possible that implementing genotype-guided approaches in daily practice for healthcare centers without these facilities will be challenging. Moreover, due to limited availability, higher cost, and contraindications, the clinical introduction of agents, such as prasugrel and ticagrelor, is still low in some regions [33]. However, results from this meta-analysis provide robust evidence showing the net benefit of efficacy, which easily tilts the scale of medication cost for every stroke, in-stent thrombosis, or MI case that subsequently needs hospitalization or repeat entrance into the health care with more imaging and tests which can increase the total cost of treatment.

The results of our meta-analysis should be interpreted in context of several limitations. First, this meta-analysis was performed under the assumption that the baseline characteristics of the patients in the included trials were adequately similar. Discrepancies in patient characteristics and background therapy could have possibly contributed to clinical heterogeneity; however, a low statistical heterogeneity was noted in six of our eight included outcomes. Second, treatment options including the type of stent used during PCI differed among the included studies, which can have an impact on the time duration of the usage of the P2Y_12_ inhibitor. Third, follow-up durations varied throughout the included trials which might have potentially impacted the results. The interpretation of previous studies has been limited by small sample sizes but through this meta-analysis and subgroup analyses, we were able to pool the results and differentiate the results between genetic *vs* platelet function testing, and escalation *vs* de-escalation strategy.

## 5 Conclusion

Individualized guided selection of antiplatelet therapy significantly reduced the incidence of MACE, MI, ST, stent thrombosis, stroke, and minor bleeding in adult patients when compared with standard antiplatelet therapy. Our findings support the implementation of genetic testing and platelet function testing in opting for the most beneficial antiplatelet agent, maximizing patient safety, and reducing costs. Future studies considering patient-level meta-analysis would help augment the present available evidence and verify our results.

## Provenance and peer review

Not commissioned, externally peer-reviewed.

## Sources of funding

None to declare.

## Ethical approval

N/A.

## Consent

N/A.

## Authors contribution

Naser Yamani: Conceptualization, Methodology.

Samuel Unzek: Data curation and Software.

Muhammad Hasnain Mankani: Writing.

Kaneez Fatima: Reviewing.

Adeena Musheer and Talal Almas: Software, Validation.

Humera Qamar: Visualization and Investigation.

Shausha Farooq and Waqas Shahnawaz: Editing.

Vincent Figuerdo: Supervision.

Farouk Mookadam: Reviewing.

## Registration of research studies

1. Name of the registry:
2. Unique Identifying number or registration ID:
3. Hyperlink to your specific registration (must be publicly accessible and will be checked):

## Guarantor

Talal Almas Talalalmas.almas@gmail.com.

## Declaration of competing interest

None to declare.
