# Supplementary material for: Does individualized guided selection of antiplatelet therapy improve outcomes after percutaneous coronary intervention? A systematic review and meta-analysis
Source: Ann Med Surg (Lond). 2022 Jun 18;79:103964. doi: 10.1016/j.amsu.2022.103964 (PMC9289299; doi:10.1016/j.amsu.2022.103964)
Supplement: Multimedia component 1 [file mmc1.docx]

**Supplementary Appendix**

**Supplementary table 1: Detailed search strategy for each of the included databases.**

**Supplementary table 2: Quality Assessment of Randomized Controlled Trials with Cochrane Risk-of-Bias Tool.**

**Supplementary figure 1: Funnel plot assessing publication bias.**

**Supplementary figure 2: Forest plot comparing guided antiplatelet therapy with standard antiplatelet therapy for major adverse cardiovascular events.**

**Supplementary figure 3: Forest plot comparing guided antiplatelet therapy with standard antiplatelet therapy for cardiovascular mortality.**

**Supplementary figure 4: Forest plot comparing guided antiplatelet therapy with standard antiplatelet therapy for all-cause mortality.**

**Supplementary figure 5: Forest plot comparing guided antiplatelet therapy with standard antiplatelet therapy for myocardial infarction.

Supplementary figure 6: Forest plot comparing guided antiplatelet therapy with standard antiplatelet therapy for stent thrombosis.

Supplementary figure 7: Forest plot comparing guided antiplatelet therapy with standard antiplatelet therapy for stroke.**

**Supplementary figure 8: Forest plot comparing guided antiplatelet therapy with standard antiplatelet therapy for major bleeding.**

**Supplementary figure 9: Forest plot comparing guided antiplatelet therapy with standard antiplatelet therapy for minor bleeding.**

Supplementary table 1: Detailed search strategy for each of the included databases.

| Database | Search Strategy | Articles retrieved |
| --- | --- | --- |
| MEDLINE | ("phenotype"[MeSH Terms] OR "phenotype"[All Fields] OR "phenotypes"[All Fields] OR "phenotyped"[All Fields] OR "phenotypic"[All Fields] OR "phenotypical"[All Fields] OR "phenotypically"[All Fields] OR "phenotyping"[All Fields] OR "phenotypings"[All Fields] OR ("genotype"[MeSH Terms] OR "genotype"[All Fields] OR "genotypes"[All Fields] OR "genotypic"[All Fields] OR "genotype s"[All Fields] OR "genotyped"[All Fields] OR "genotyper"[All Fields] OR "genotypical"[All Fields] OR "genotypically"[All Fields] OR "genotyping"[All Fields] OR "genotypings"[All Fields] OR "genotypization"[All Fields])) AND ("clopidogrel"[MeSH Terms] OR "clopidogrel"[All Fields] OR "clopidogrel s"[All Fields] OR ("antiplatelet"[All Fields] OR "antiplatelets"[All Fields]) OR ("percutaneous coronary intervention"[MeSH Terms] OR ("percutaneous"[All Fields] AND "coronary"[All Fields] AND "intervention"[All Fields]) OR "percutaneous coronary intervention"[All Fields])) | 1941 |
| Cochrane  Central | **(phenotype OR genotype) AND (clopidogrel OR antiplatelet OR percutaneous coronary intervention)** | 369 |

Supplementary Table 2: Quality Assessment of Randomized Controlled Trials with Cochrane Risk-of-Bias Tool.

| # | Study, year | Sequence Generation  (selection bias) | Allocation concealment (selection bias) | Blinding of Participants (performance bias) | Blinding of Outcome assessment (detection bias) | Incomplete outcome data (attrition bias) | Selective outcome reporting (reporting bias) | Other sources of bias |
| --- | --- | --- | --- | --- | --- | --- | --- | --- |
| 1 | Hazarbasanov et al, 2012 | Unclear | Low | Low | High | Low | Unclear | High |
| 2 | Collet et al, 2012  (ARCTIC) | Low | Low | Low | Low | Low | Low | Low |
| 3 | Xie et al, 2013  (IAC-PCI) | Low | Unclear | Low | High | Low | Low | High |
| 4 | Zhu et al, 2015 | Low | Low | Low | High | Low | Low | High |
| 5 | Cayla et al, 2016 (ANTARCTIC) | Low | Low | Low | Low | Low | Low | Low |
| 6 | Sibbing et al, 2017 (TROPICAL-ACS) | Low | Low | Low | Low | Low | Low | Low |
| 7 | Notarangelo et al, 2018  (PHARMCLO) | Low | Low | Low | High | Low | Low | High |
| 8 | Claassens et al, 2019  (POPular Genetics) | Low | Low | Low | Low | Low | Low | Low |
| 9 | Tuteja et al, 2020 | High | High | Low | High | Low | Low | High |
| 10 | Zheng et al, 2020  (PATH-PCI) | High | High | High | High | Low | Low | High |
| 11 | Pereira et al, 2020 (TAILOR-PCI) | Low | Low | Low | Low | Low | Low | Low |

Supplementary figure 1: Funnel plot assessing publication bias.


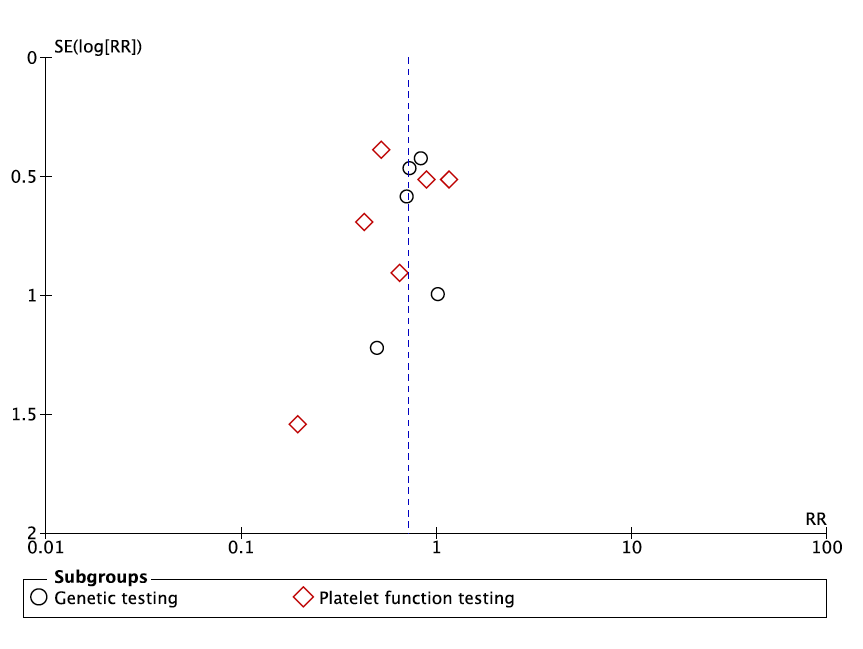


SE, standard error; RR, risk ratio

Supplementary figure 2: Forest plot comparing guided antiplatelet therapy with standard antiplatelet therapy for major adverse cardiovascular events.


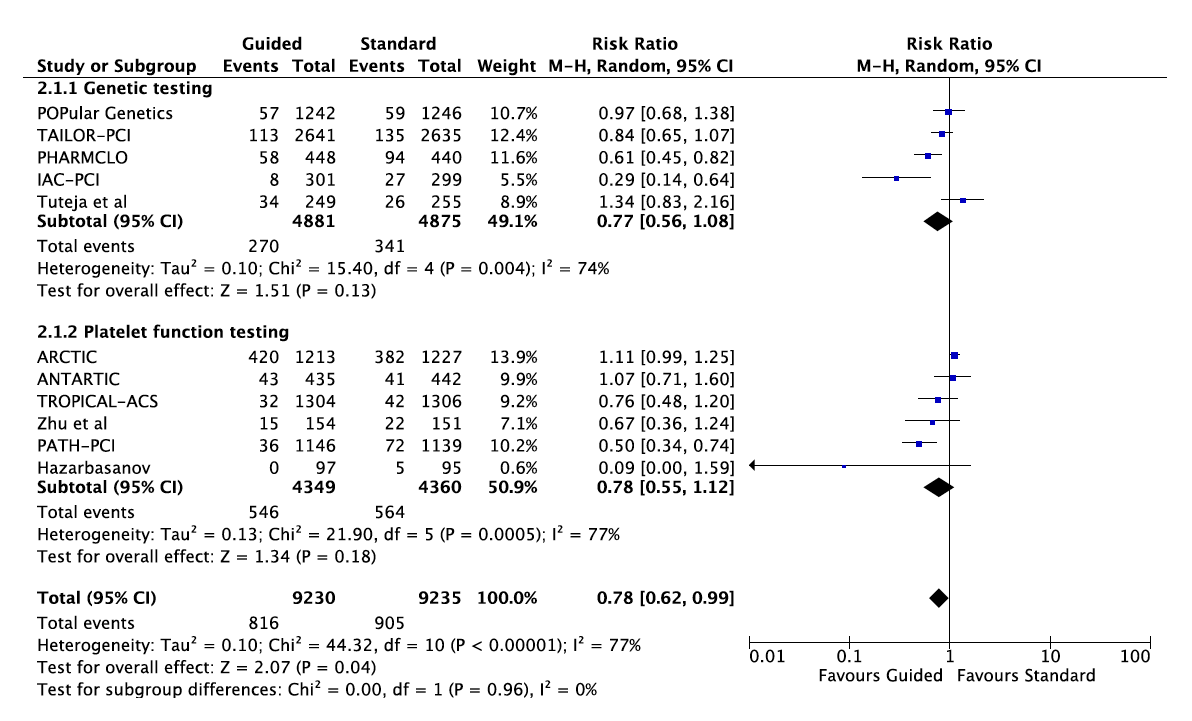


Supplementary figure 3: Forest plot comparing guided antiplatelet therapy with standard antiplatelet therapy for cardiovascular mortality.

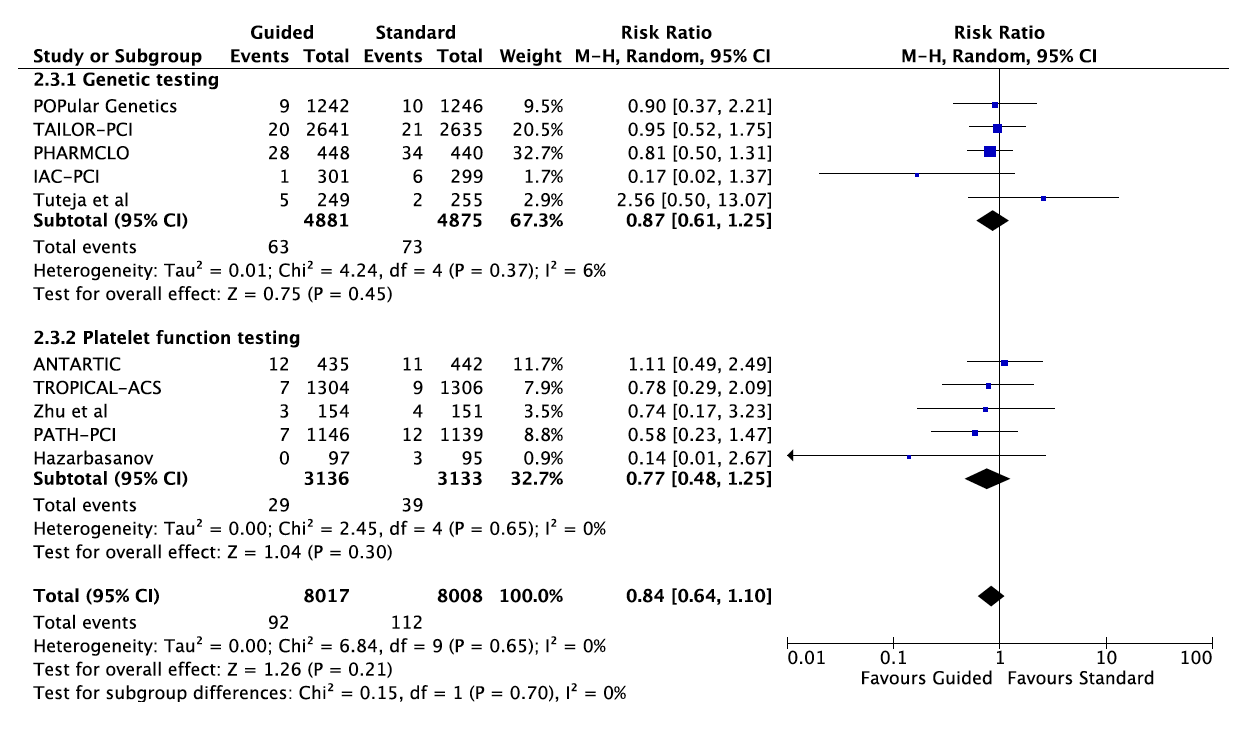


Supplementary figure 4: Forest plot comparing guided antiplatelet therapy with standard antiplatelet therapy for all-cause mortality.


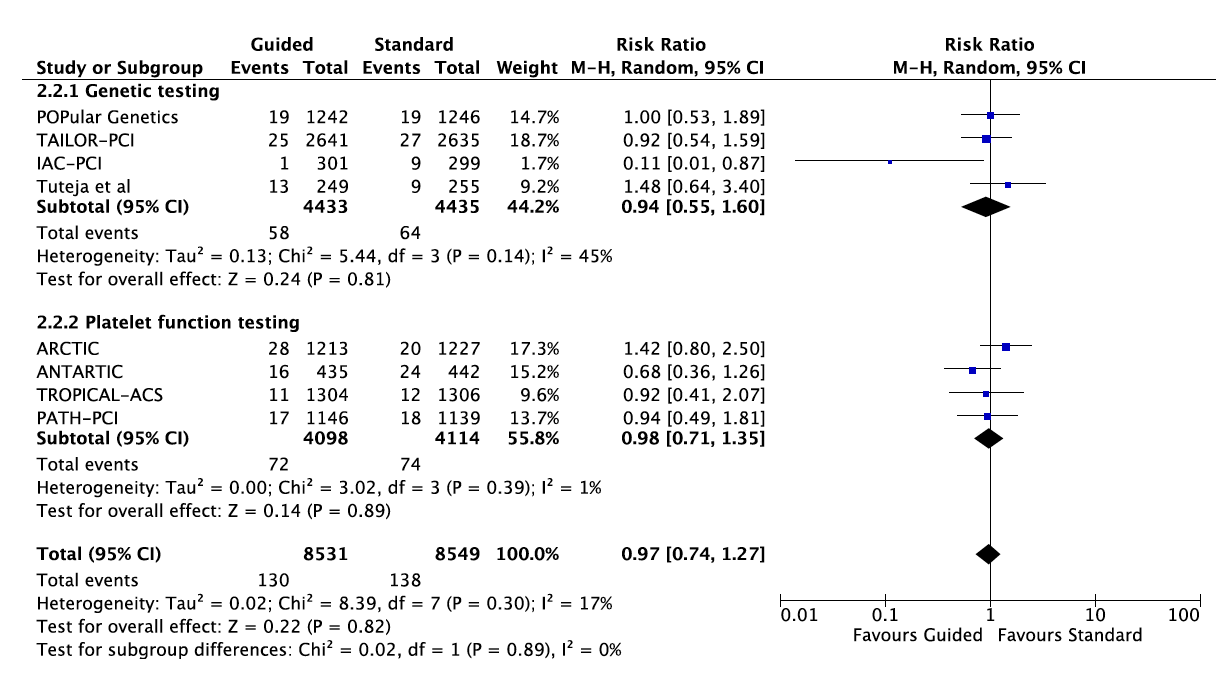


Supplementary figure 5: Forest plot comparing guided antiplatelet therapy with standard antiplatelet therapy for myocardial infarction.

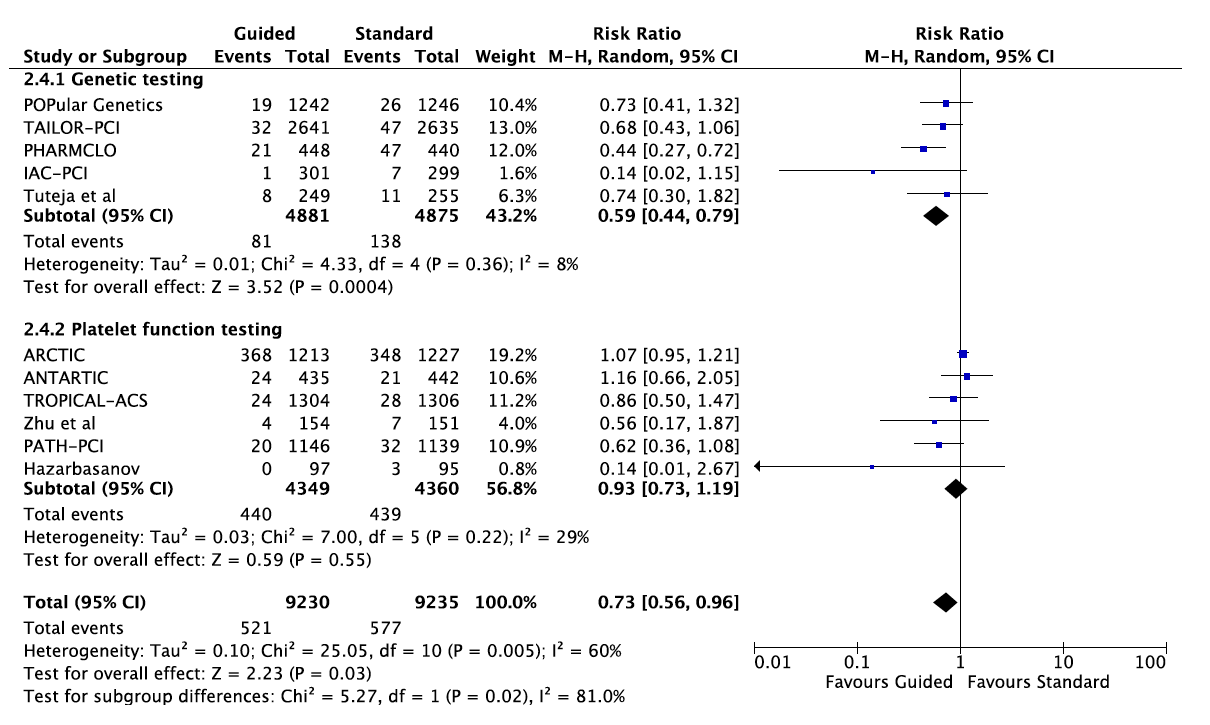


Supplementary figure 6: Forest plot comparing guided antiplatelet therapy with standard antiplatelet therapy for stent thrombosis.

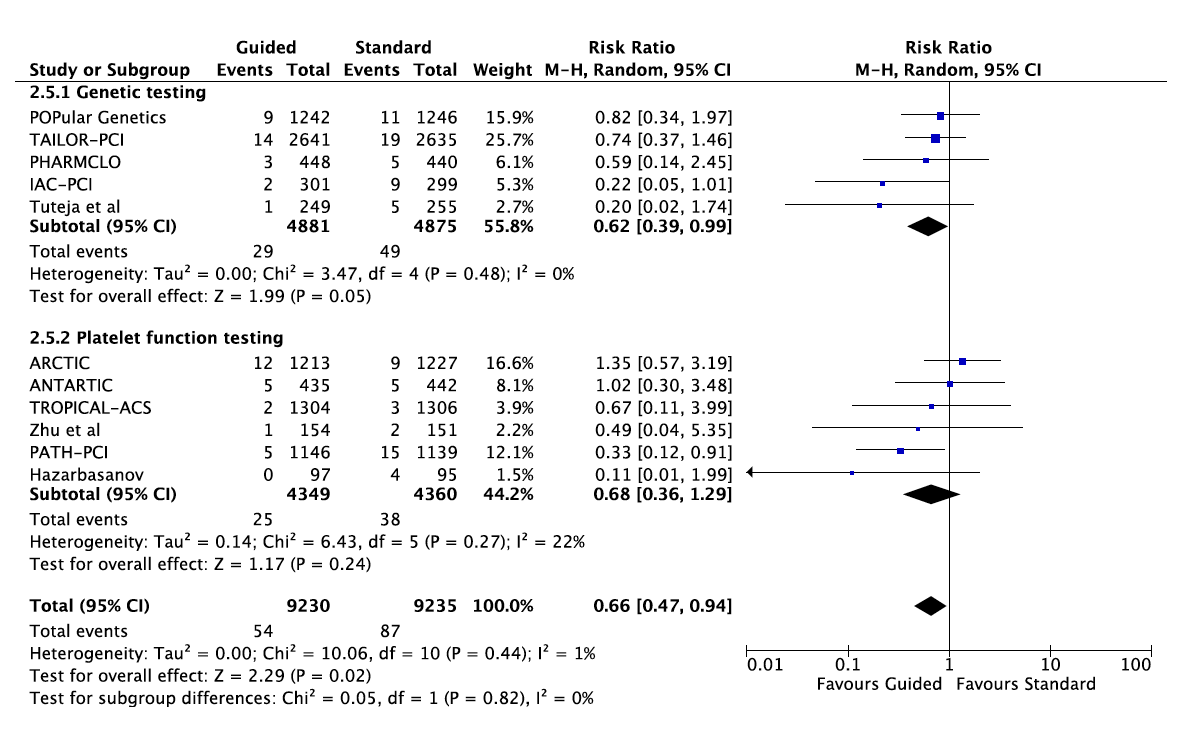


Supplementary figure 7: Forest plot comparing guided antiplatelet therapy with standard antiplatelet therapy for stroke.


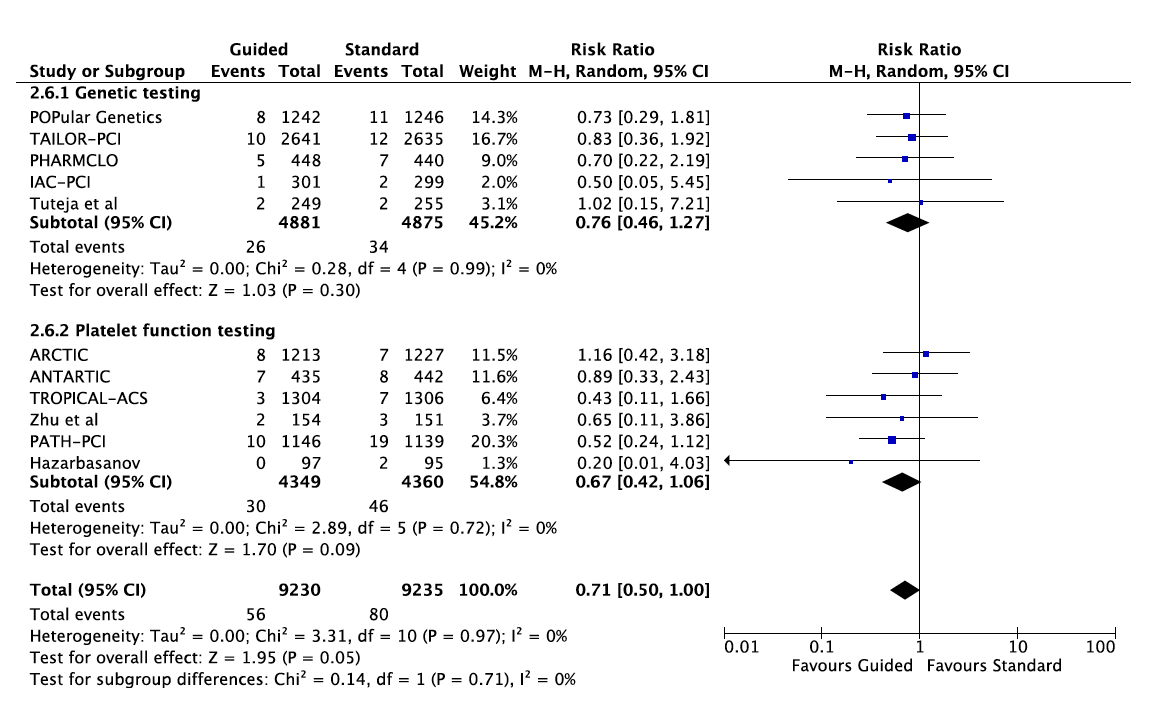


Supplementary figure 8: Forest plot comparing guided antiplatelet therapy with standard antiplatelet therapy for major bleeding.


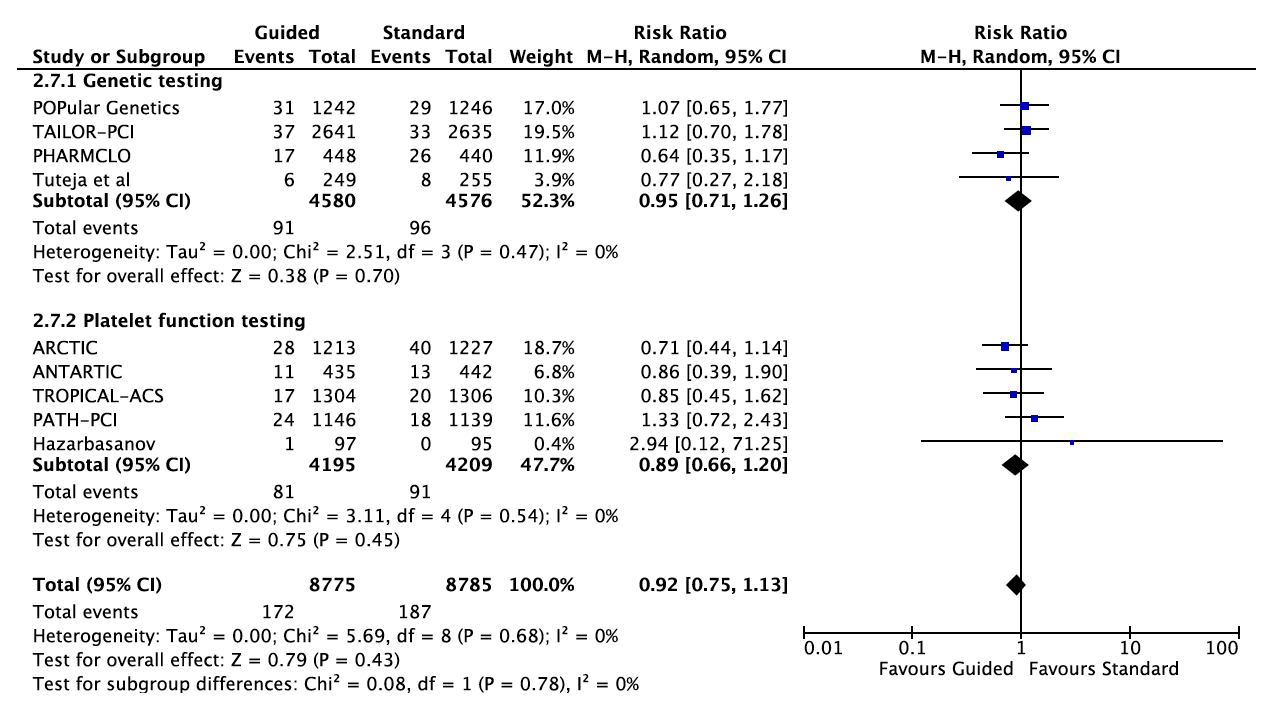


Supplementary figure 9: Forest plot comparing guided antiplatelet therapy with standard antiplatelet therapy for minor bleeding.

**
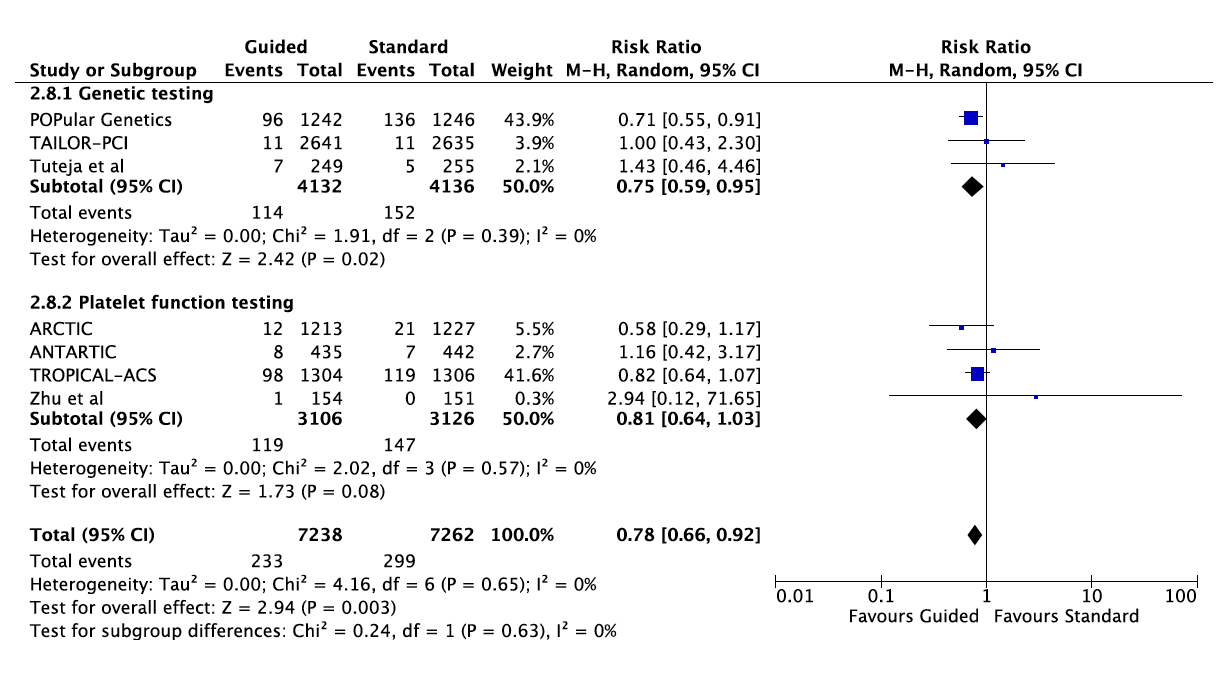
**
